# Retraction Note: Monitoring the temperature through moving average control under uncertainty environment

**DOI:** 10.1038/s41598-022-15747-w

**Published:** 2022-07-04

**Authors:** Muhammad Aslam, Abdulmohsen Al Shareef, Khushnoor Khan

**Affiliations:** 1 Department of Statistics, Faculty of Science, King Abdulaziz University, Jeddah, 21551 Saudi Arabia; 2 Deanship of Scientific Research, King Abdulaziz University, Jeddah, 21589 Saudi Arabia

Retraction of: *Scientific Reports* 10.1038/s41598-020-69192-8, published online 22 July 2020

The Editors have retracted this Article. Following publication, concerns were raised about the rationale for the approach presented and the underlying reasoning. A post-publication review of the authors' mathematical arguments revealed a lack of clarity in the terms presented and inferences that are not adequately justified. The Editors therefore no longer have confidence in the conclusions presented.

Muhammad Aslam and Khushnoor Khan do not agree to this retraction. Abdulmohsen Al Shareef did not respond to correspondence relating to this retraction.

